# Digital twins in healthcare: a comprehensive review and future directions

**DOI:** 10.3389/fdgth.2025.1633539

**Published:** 2025-11-18

**Authors:** Hamid Khoshfekr Rudsari, Becky Tseng, Hongxu Zhu, Lulu Song, Chunhui Gu, Abhishikta Roy, Ehsan Irajizad, Joseph Butner, James Long, Kim-Anh Do

**Affiliations:** 1 Department of Biostatistics, The University of Texas MD Anderson Cancer Center, Houston, TX, United States; 2 Department of Radiation Oncology, The University of Texas MD Anderson Cancer Center, Houston, TX, United States

**Keywords:** digital twin, personalized medicine, healthcare modeling, predictive healthcare, AI in medicine, patient-specific models, virtual simulation, computational medicine

## Abstract

Digital Twin (DT) technology has emerged as a transformative force in healthcare, offering unprecedented opportunities for personalized medicine, treatment optimization, and disease prevention. This comprehensive review examines the current state of DTs in healthcare, analyzing their implementation across different physiological levels—from cellular to whole-body systems. We systematically review the latest developments, methodologies, and applications while identifying challenges and opportunities. Our analysis encompasses technical frameworks for cardiovascular, neurological, respiratory, metabolic, hepatic, oncological, and cellular DTs, highlighting significant achievements such as population-scale cardiac modeling (3,461 patient cohort), reduced atrial fibrillation recurrence rates through patient-specific cardiac models, improved brain tumor radiotherapy planning, advanced liver regeneration modeling with real-time simulation capabilities, and enhanced glucose management in diabetes. We detail the methodological foundations supporting different DT implementations, including data acquisition strategies, physics-based modeling approaches, statistical learning algorithms, neural network-based control systems, and emerging artificial intelligence techniques. While discussing implementation challenges related to data quality, computational constraints, and validation requirements, we provide a forward-looking perspective on future opportunities for enhanced personalization, expanded application areas, and integration with emerging technologies. This review offers a multidimensional assessment of healthcare DTs and outlines future directions for their development and integration. This review demonstrates that while healthcare DTs have achieved remarkable clinical successes—from reducing cardiac arrhythmia recurrence rates by over 13% to enabling 97% accuracy in neurodegenerative disease prediction, and achieving sub-millisecond liver response predictions with high accuracy—their clinical translation requires addressing challenges such as data integration, computational scalability, digital equity, and validation frameworks.

## 1 Introduction

Digital twin (DT), a concept first introduced by Grieves in 2002 as a “conceptual ideal” for product life cycle management, defines the triad of (i) a physical system, (ii) its virtual representation, and (iii) the bilateral information flow that links the physical and the virtual counterparts together [1]. This framework bridges the physical and digital realms, enabling the analysis of past and present processes and facilitating future predictions [2]. Initially developed for manufacturing and aerospace industries [3], DT technology has rapidly evolved to meet the critical demands of modern healthcare.

In clinical applications, DTs facilitate personalized medicine by enabling the construction of patient-specific models [4]. These models integrate data from electronic health records (EHR), imaging modalities, and Internet of Things (IoT) devices to account for individual physiological and historical nuances [5]. Such comprehensive models empower clinicians to tailor treatment strategies for each patient, optimizing therapeutic interventions and improving clinical outcomes [6]. Moreover, by providing a virtual environment for simulation, DTs allow for risk-free experimentation, where various treatment scenarios can be tested and refined before actual clinical application. This not only minimizes potential risks associated with trial-and-error approaches but also contributes to significant cost reductions in healthcare delivery.

The real-time monitoring capacity of DTs further enhances their impact: by continuously updating the digital replicas with new patient data, healthcare providers can anticipate and respond to emerging health issues promptly, thereby reducing the incidence of critical complications [7]. In the realm of precision cardiology, for example, digital heart models have been successfully employed to simulate interventions and guide surgical planning, ultimately reducing procedure-related risks and optimizing patient-specific treatment plans [8]. In addition, DTs can predict the progression of diseases and recommend preventive measures, thereby enabling timely interventions and further enhancing patient outcomes[9]. Additionally, by streamlining operational processes such as resource allocation and predictive maintenance of medical devices, DTs directly address the escalating healthcare costs and inefficiencies inherent in traditional care delivery models [10].

### 1.1 Importance of digital twins in healthcare

DTs represent a paradigm shift in healthcare delivery and medical research, offering virtual replicas of physical entities that can be used for simulation, prediction, and optimization. The importance of DTs in healthcare stems from several key factors:

- **Personalized medicine**: DTs enable highly individualized treatment approaches by creating patient-specific models that account for unique physiological characteristics and medical histories. These models integrate multi-omics data, clinical parameters, and lifestyle factors to create comprehensive patient profiles that guide precision therapeutics and interventions [4, 11, 12]. By capturing individual variability in genes, environment, and lifestyle,DTs facilitate the realization of the P4 medicine paradigm;predictive, preventive, personalized, and participatory healthcare [13].
- **Real-time monitoring**: DT’s provide continuous monitoring and analysis of patient health status, enabling early detection of potential health issues and timely interventions. Advanced DTs incorporate data from wearable sensors, implantable devices, and ambient monitoring systems to create dynamic models that evolve with the patient’s condition. This continuous feedback loop allows for the detection of subtle physiological changes that might precede clinical manifestations of disease by days or weeks, creating opportunities for preemptive interventions [14–16].
- **Risk-free experimentation**: DTs allow healthcare providers to simulate different treatment scenarios without risking patient safety, optimizing treatment plans before implementation [17]. Clinicians can evaluate multiple therapeutic approaches, drug dosages, and intervention timings on the digital replica before applying them to the actual patient. This capability is particularly valuable in complex cases involving multimorbidity, where treatment interactions and compound effects are difficult to predict. In surgical planning, DTs enable surgeons to rehearse procedures on patient-specific anatomical models, anticipate complications, and optimize technical approaches, resulting in reduced operative times and improved outcomes [18].
- **Cost reduction**: By enabling virtual testing and optimization, DTs can significantly reduce healthcare costs associated with trial-and-error approaches in treatment. Economic analyses suggest that implementation of DT technology could reduce hospital readmission rates by up to 25% for certain chronic conditions through improved treatment planning and patient monitoring [19, 20]. Furthermore, DTs optimize resource utilization by predicting patient flow, equipment needs, and staffing requirements, thereby reducing operational inefficiencies. The long-term economic benefits extend to reduced disability costs, fewer complications, and shortened hospital stays, collectively contributing to more sustainable healthcare systems.
- **Enhanced decision support**: DTs serve as sophisticated clinical decision support systems that augment human expertise with computational precision. By integrating machine learning (ML) algorithms and causal inference models, DTs can identify patterns and correlations in patient data that might escape human observation. This capability transforms the decision-making process from intuition-based to evidence-driven, particularly in complex clinical scenarios where multiple factors must be considered simultaneously [21, 22]. The transparent nature of well-designed DTs also allows clinicians to understand the reasoning behind recommendations, facilitating informed clinical judgment.
- **Longitudinal health management**: DTs enable lifetime health monitoring and management by maintaining a dynamic virtual representation of an individual’s health status across their lifespan. This longitudinal perspective supports preventive healthcare strategies by identifying risk trajectories and intervention opportunities long before disease manifestation. For chronic disease management, DTs provide a cohesive framework that integrates episodic care events into a continuous care model, enhancing treatment consistency and long-term outcomes [23, 24].

### 1.2 Applications in healthcare

**Figure 1:**
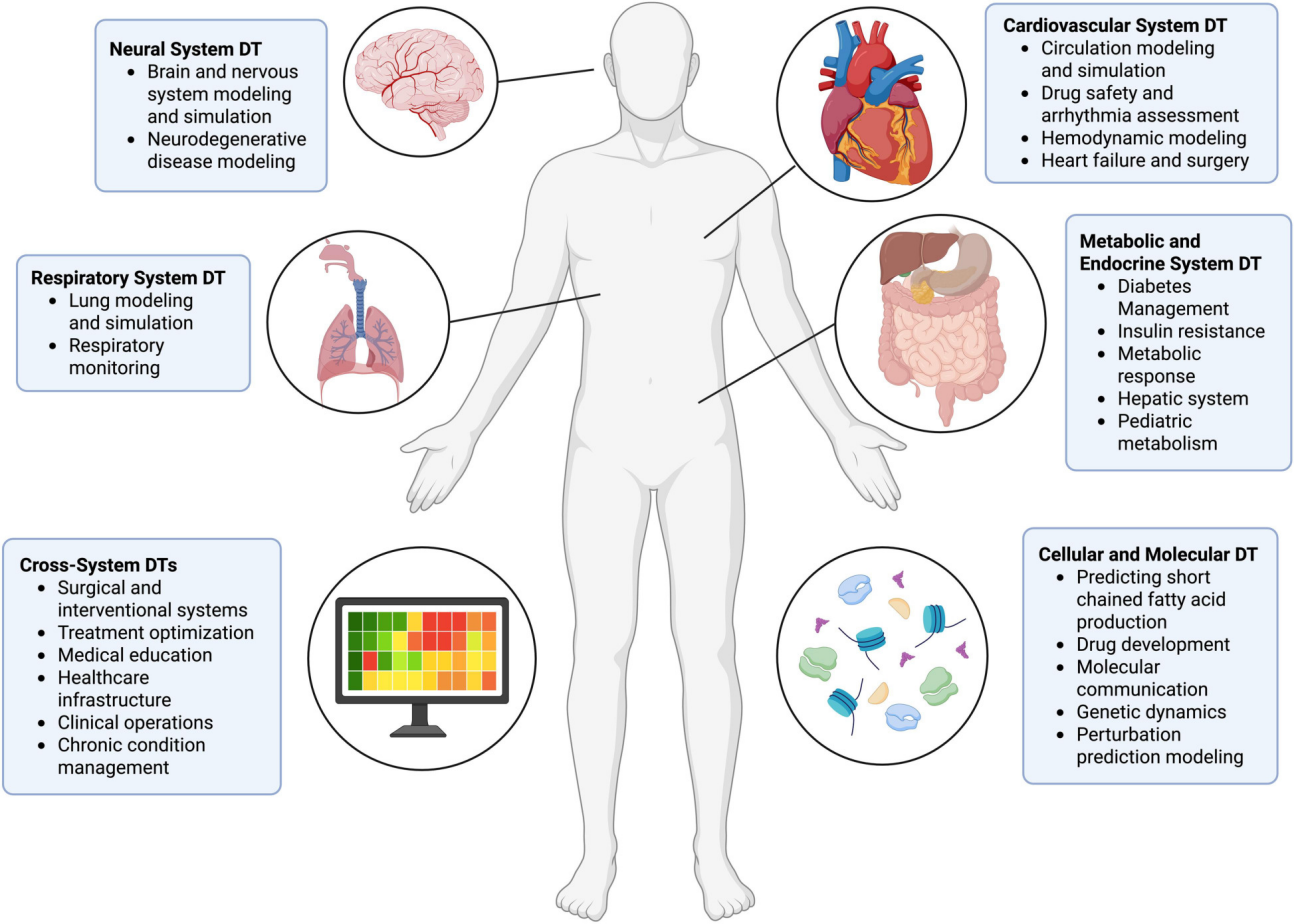
Digital twin models in this review. The illustration is created using BioRender.com.

As shown in Figure 1, the applications of DTs in healthcare span across various domains, as evidenced by the numerous implementations throughout medical specialties:

- **Cardiovascular applications**: DTs have transformed cardiac care through applications ranging from molecular-level drug interaction studies to organ-level hemodynamic simulations. For drug safety assessment, DTs can predict pro-arrhythmic risks with remarkable concordance with clinical observations, as demonstrated in studies evaluating hydroxychloroquine and azithromycin [25]. Patient-specific cardiac DTs have shown clinical utility in guiding antiarrhythmic drug selection, with studies demonstrating significantly lower recurrence rates (40.9% vs. 54.1%) when treatment was guided by virtual testing [26]. In hemodynamic monitoring, the longitudinal hemodynamic mapping framework (LHMF) has achieved unprecedented accuracy with error rates between 0.0002%–0.004% for simulating hundreds of heartbeats [27], while the Cardio Twin architecture provides real-time electrocardiogram (ECG) monitoring with 85.77% classification accuracy and 95.53% precision [28]. For surgical applications, digital hearts have revolutionized procedures like ventricular tachycardia ablation by incorporating tissue characteristics into 3D models, achieving significant reductions in ablation volumes while maintaining high concordance with clinical outcomes [29].
- **Neurological applications**: In neurology, DTs have enabled unprecedented insights into disease progression and treatment planning. Physics-based models integrating the Fisher-Kolmogorov equation with anisotropic diffusion have successfully simulated the spread of misfolded proteins across the brain, capturing both spatial and temporal aspects of neurodegenerative disease progression [30]. For multiple sclerosis (MS), DTs have revealed that progressive brain tissue loss begins on average 5–6 years before clinical symptom onset [31]. Parkinson’s disease management has been enhanced through DT-based Healthcare Systems achieving prediction accuracy of 97.95% for earlier identification from remote locations [32]. For brain tumors, hybrid approaches combining Semi-Supervised Support Vector Machine (S3VM) and improved AlexNet CNN have achieved feature recognition accuracy of 92.52% with impressive segmentation metrics [33], while personalized radiotherapy planning for high-grade gliomas has demonstrated either increased tumor control or significant reductions in radiation dose (16.7%) while maintaining equivalent outcomes [34].
- **Respiratory system applications**: DTs of the respiratory system integrate multiple scales of analysis, from alveolar mechanics to whole-organ function, enabling detailed simulation of lung biomechanics in both health and disease states [35]. These models facilitate personalized treatment strategies and improved understanding of structure-function relationships. For non-invasive monitoring, systems using ESP32 Wi-Fi Channel State Information sensors have achieved 92.3% accuracy in breathing rate estimation, while ML techniques have demonstrated classification accuracies of 89.2% for binary-class and 83.7% for multi-class respiratory pattern recognition [36]. For lung cancer management, the DT-GPT model forecasts clinical variables with high accuracy ($R^{2}$ of 0.98), while the Lung-DT framework employs YOLOv8 neural networks to classify chest X-rays with exceptional performance (96.8% accuracy, 92% precision) [37].
- **Metabolic and endocrine applications**: DTs for diabetes management have demonstrated remarkable clinical utility. The Exercise Decision Support System (exDSS) for type 1 diabetes (T1D) provides personalized recommendations during exercise, increasing time in target glucose range from 80.2% to 92.3% and reducing hypoglycemia incidents from 15.1% to 5.1% during aerobic activities [38]. For type 2 diabetes, comprehensive frameworks combining ML, multiomic data, and knowledge graphs have enhanced predictive accuracy for disease trajectories and treatment responses [39]. Multi-scale DTs for adiposity-driven insulin resistance successfully integrate mechanistic models of glucose metabolism, body composition, and cellular insulin signaling to predict responses to dietary and pharmacological interventions [40]. Specialized pediatric models, such as sex-specific, personalized metabolic whole-body models for newborns and infants, demonstrate strong agreement with World Health Organization (WHO) growth standards while providing insights into early-life metabolism and disease progression [41].
- **Oncology applications**: DTs have revolutionized cancer care across multiple dimensions. For prostate cancer, ML-based systems have achieved 96.25% accuracy in biochemical recurrence prediction [42], while AI-based DTs of pathologists have demonstrated comparable performance to human experts in detecting cancer and estimating tumor volume [43]. In head and neck cancers, DT technology employing deep Q-learning has improved survival rates by 3.73% and reduced dysphagia rates by 0.75% for oropharyngeal squamous cell carcinoma [44], while AI-directed frameworks measuring soft tissue shift during surgery have enabled precise volume measurements with applications in frozen section management and improved surgical precision [45]. The PRIMAGE project for pediatric oncology integrates imaging biomarkers, clinical data, and artificial intelligence (AI), achieving high accuracy in tumor segmentation (Dice similarity coefficient of 0.997) while reducing radiologist workload by 93% [46]. At the tumor microenvironment level, DTs investigating mechanical stresses and immune surveillance have revealed critical insights into tumor behavior, invasive phenotypes, and potential immunotherapy strategies [47, 48].
- **Cellular and molecular applications**: At the cellular level, DTs have enabled unprecedented insights into metabolic regulation and cellular behavior. Perturbation prediction models simulate cellular responses to drugs, gene knockouts, or metabolic changes, accelerating research in drug discovery by enabling in silico experiments that significantly reduce costs and resource requirements [49, 50]. Advanced approaches include differential equation-based dynamic models like Bicycle [51] and Cellbox [52] that capture gene-regulatory networks and cellular interactions, as well as deep learning models like scGPT [53] that treat gene expression profiles as “sentences” and individual genes as “tokens” to predict genetic perturbation responses. For drug development, DTs have demonstrated higher accuracy (89%) than traditional animal models (75%) in predicting cardiac pro-arrhythmic cardiotoxicity [54], while comprehensive frameworks address drug resistance in cancer treatment by incorporating both irreversible and reversible resistance mechanisms [55].
- **Clinical operations and healthcare infrastructure**: DTs have transformed healthcare delivery systems and operational efficiency. Standardized frameworks compliant with ISO/IEEE 11073 standards have integrated health devices for population monitoring with classification accuracy up to 96.85% [56]. Comprehensive DT ecosystems for oncology clinical operations incorporate specialized twins for medical necessity evaluation, care navigation, and clinical history visualization, streamlining workflows and enhancing decision-making [57]. Early Warning Systems leveraging DT technology have reduced code blue incidents by 60% through predictive analytics and early intervention [58]. For clinical trials, innovations like ClinicalGAN create patient DTs that outperform state-of-the-art approaches by 3%–4% in generation quality metrics and demonstrate 5%–10% improvement in patient drop-off prediction [16], while TWIN-GPT establishes cross-dataset associations despite limited data availability, boosting clinical trial outcome prediction beyond previous approaches [59].
- **Surgical and interventional applications**: DT technology has revolutionized surgical planning and execution across specialties. Novel digital-twin-enabled Internet of Medical Things (IoMT) systems for telemedical surgical simulation integrate mixed reality, 5G cloud computing, and deep learning techniques, achieving 92%–93% predictive accuracy for complex surgical scenarios [60]. For minimally invasive approaches, DTs of magnetic medical microrobots incorporate stochastic Model Predictive Control enhanced by ML, demonstrating high precision in navigating complex biological environments for targeted drug delivery [61]. Patient-specific, three-dimensional mixed-reality anatomical models derived from CT or MRI data enable surgeons to interact with highly accurate representations of anatomy, improving understanding of complex structures and spatial relationships while offering superior visualization compared to conventional methods [62].

Table 1 and Figure 1 provide a comprehensive overview of the DT applications across different healthcare domains and organs discussed in this review. The table highlights key findings and performance metrics from studies in cardiovascular, neurological, respiratory, metabolic, hepatic, oncological, cellular, and cross-system applications, demonstrating the breadth and depth of DT technology in modern healthcare.

**Table 1:** Summary of DT applications across healthcare domains.

Domain	Application area	Key findings/contributions	References
Cardiovascular	Drug safety assessment	DTs predict pro-arrhythmic risks with 89% accuracy, outperforming animal models (75%). Patient-specific virtual drug testing reduced AF recurrence rates (40.9% vs 54.1%).	[25, 26, 54, 63, 64]
	Hemodynamic modeling	LHMF framework achieved error rates of 0.0002%–0.004% for simulating cardiac cycles. Cardio Twin architecture provided 85.77% classification accuracy with 95.53% precision.	[27, 28]
	Surgical applications	DIFAT approach for VT ablation reduced volumes (1.87 ${\mathrm{cm}}^{3}$ vs. 7.05 ${\mathrm{cm}}^{3}$) while maintaining 79% overlap with clinical targets.	[29]
	Population studies	Large-scale cardiac DTs (3,461 from UK Biobank) revealed sex-specific QRS differences explained by anatomy. Sex-dependent drug classification achieved >89% accuracy for antiarrhythmic drugs.	[65, 68]
	Monitoring frameworks	TwinCardio framework provides comprehensive cardiovascular monitoring through IoT integration with customized neural networks for disease classification.	[66]
Neurological	Neurodegenerative disease	Physics-based models simulate protein misfolding propagation. MS DTs revealed brain atrophy begins 5–6 years before symptoms. Parkinson’s DTHS achieved 97.95% prediction accuracy.	[30–32, 69]
	Brain tumor analysis	Hybrid S3VM-AlexNet achieved 92.52% feature recognition accuracy. Personalized radiotherapy planning reduced radiation dose by 16.7% while maintaining outcomes.	[33, 34]
	Early diagnosis	DADD digital twin model achieved 88% accuracy in identifying CSF biomarker positivity and 87% accuracy in predicting clinical conversions using non-invasive EEG recordings.	[70]
Respiratory	Lung modeling	Multi-scale integration from alveolar to organ-level mechanics enabling detailed simulation of lung biomechanics.	[35]
	Respiratory monitoring	ESP32 Wi-Fi sensors achieved 92.3% accuracy in breathing rate estimation. ML techniques demonstrated 89.2% binary-class accuracy.	[36]
Metabolic & endocrine	Diabetes management	exDSS increased time in target glucose range from 80.2% to 92.3% during exercise. Type 2 diabetes framework enhanced predictive accuracy through multiomic data integration.	[38, 39, 71]
	Insulin resistance	Multi-scale DTs integrated glucose metabolism, body composition, and cellular insulin signaling to predict intervention responses.	[40]
	Pediatric metabolism	Sex-specific models for newborns and infants showed strong agreement with WHO growth standards.	[41]
Hepatic	Liver regeneration	Mathematical mechanism-based model captured complex microarchitecture and cellular interactions during regeneration.	[73]
	Real-time simulation	Thermodynamics-informed graph neural networks achieved liver response prediction in 1.65 ms with <0.15% position errors and <7% stress estimation errors.	[74]
	Organ-on-chip	DigiLoCS platform successfully predicted liver clearance for 32 drugs with superior performance compared to conventional models through comprehensive ODE-based modeling.	[75]
Oncology	Prostate cancer	ML-based system achieved 96.25% accuracy in biochemical recurrence prediction. AI-based DTs performed comparably to human pathologists.	[42, 43]
	Lung cancer	DT-GPT forecasted clinical variables with $R^{2}$ of 0.98. Lung-DT classified chest X-rays with 96.8% accuracy and 92% precision.	[37, 79]
	Head & neck cancer	Deep Q-learning improved survival rates by 3.73% and reduced dysphagia by 0.75% for OPSCC. AI-directed framework measured soft tissue shift during surgery.	[44, 45]
	Tumor microenvironment	DTs revealed insights into mechanical stresses, tumor behavior, and immune surveillance of micrometastases.	[47, 48]
	Radiation therapy	Mechanistic multiscale prostate cancer model demonstrated sensitivity to key biological parameters for personalized radiation therapy optimization.	[78]
	Specialized populations	Geriatric breast cancer tool achieved 0.81 AUC for prognostic clustering using AI and clinical-biological features.	[81]
Cellular & molecular	Perturbation prediction	Models like Bicycle and CellBox simulate cellular responses to drugs, gene knockouts, and metabolic changes, enabling in silico experiments.	[49–52, 107]
	Metabolic modeling	Community-scale models for gut microbiome achieved strong correlations (*r* = 0.62–0.63) with clinical health markers.	[82, 83]
	Molecular communication	3D partial differential equation models for normal and anomalous diffusion of extracellular vesicles achieved high accuracy in predicting biodistribution patterns and transport dynamics. DTs model mitochondrial fission through biophysical interactions, identifying protein-binding interventions. Models of endocytosis reveal how geometric instabilities drive vesicle formation.	[84–87]
Cross-system	Surgical systems	IoMT system for telemedical surgical simulation achieved 92%–93% predictive accuracy. DTs of magnetic microrobots enhanced precision in complex environments.	[60, 61]
	Clinical operations	Standardized frameworks achieved up to 96.85% classification accuracy. EWS reduced code blue incidents by 60%.	[56–58]
	Clinical trials	ClinicalGAN outperformed state-of-the-art by 3%–4% in quality metrics and demonstrated 5%–10% improvement in patient drop-off prediction.	[16, 95]
	LLM integration	TWIN-GPT established cross-dataset associations for enhanced clinical trial predictions. ScFoundation and scGPT achieved state-of-the-art performance in single-cell perturbation prediction. LLM-enabled rare tumor DTs integrated 655 publications for personalized treatment plans.	[53, 59, 97, 98]

### 1.3 Our contributions

This review makes several significant contributions to the field:

- **Comprehensive analysis**: We provide a thorough examination of current DT implementations across different physiological levels, including molecular, cellular, organ, and whole-body systems. Our analysis systematically categorizes existing approaches based on their scale, complexity, and integration capabilities, offering a structured taxonomy that clarifies the current landscape of healthcare DTs. We specifically address how different DT implementations address the critical requirements of fidelity, interoperability, and clinical relevance, providing a multidimensional assessment framework that goes beyond simple categorization.
- **Methodological framework**: We present a structured analysis of various methods used in building and implementing DTs, including data acquisition strategies, modeling approaches, validation techniques, and deployment architectures. Our framework delineates the mathematical foundations supporting different DT implementations, from statistical learning algorithms to mechanistic modeling approaches. We provide detailed assessment of computational requirements, data privacy solutions, and integration protocols that enable effective DT implementation across diverse healthcare settings. This methodological analysis serves as both an educational resource for newcomers to the field and a reference for experienced researchers seeking to optimize their DT development approaches.
- **Critical evaluation**: We offer detailed analysis of the advantages and limitations of reviewed approaches, providing an objective assessment of current technological capabilities against clinical requirements. Our evaluation incorporates multiple perspectives, including technical feasibility, clinical utility, implementation challenges, and ethical considerations, providing a balanced view of the current state of the art. We highlight specific gaps between theoretical capabilities and practical implementations, identifying key bottlenecks in computational efficiency, data availability, model validation, and clinical workflow integration that must be addressed to advance the field. This critical analysis extends to regulatory considerations and standardization needs that will influence the trajectory of DT adoption in healthcare settings.
- **Future directions**: We present a detailed roadmap for future research in DT applications for various diseases, identifying specific technological advances needed to overcome current limitations. Our forward-looking analysis outlines emerging opportunities in multimodal data integration, explainable AI, federated learning architectures, and human-computer interaction design that will shape next-generation DTs. We propose concrete research priorities for different disease domains, considering their unique modeling challenges and clinical impact potential. Additionally, we outline interdisciplinary collaboration models that can accelerate progress by leveraging complementary expertise across computational science, medicine, engineering, and ethics. The roadmap also addresses scalability considerations for transitioning promising research prototypes to widely deployed clinical tools.

In light of these expanding applications and technological advancements, this review synthesizes the current landscape of DTs in healthcare while providing a structured framework for understanding their development and implementation. We begin by examining DT applications across major physiological systems—cardiovascular, neural, respiratory, metabolic and endocrine, hepatic, and cellular—followed by cross-system implementations that address broader clinical needs. For each domain, we analyze the technological approaches, clinical outcomes, and remaining challenges. We then explore the methodological foundations of healthcare DTs, including data collection strategies, modeling approaches (physics-based, statistical, and AI-driven), and system integration techniques. By critically evaluating the strengths and limitations of current implementations, we identify key research gaps and technological barriers that must be addressed. Finally, we present a forward-looking perspective on the evolution of healthcare DTs, outlining promising research directions and emerging applications that will shape the future of personalized, predictive medicine. Through this comprehensive analysis, we aim to provide researchers, clinicians, and technology developers with a roadmap for advancing DT technology toward its full potential as a transformative force in healthcare.

## 2 Cardiovascular system digital twins

Recent advancements in cardiovascular DTs have revolutionized our approach to cardiac care through various sophisticated applications. These developments span from molecular-level drug interaction studies to organ-level hemodynamic simulations, demonstrating the versatility of DT technology in cardiology. The integration of high-performance computing with biological modeling has enabled unprecedented accuracy in predicting cardiac responses to various interventions.

### 2.1 Drug safety and arrhythmia assessment

Researchers developed a high-performance computational framework for in-silico cardiac trials incorporating sex-specific ion channel characteristics and phenotypic variability in 3D heart models [25]. The framework assessed hydroxychloroquine and azithromycin pro-arrhythmic risks, achieving 21.8% risk prediction for hydroxychloroquine with remarkable clinical concordance. The model incorporated electrophysiology simulations at cycle lengths of 600 ms and 400 ms, capturing cardiotoxic responses within 24 h.

DT technology expanded this approach for cardiovascular modeling and drug testing [63]. Hwang et al. demonstrated virtual antiarrhythmic drug tests in atrial fibrillation patients post-catheter ablation, showing lower recurrence rates (40.9% vs. 54.1%) with DT-guided therapy [26]. Virtual amiodarone testing revealed AF recurrence rates of 20.8% in the effective group vs. 45.1% in the ineffective group (adjusted hazard ratio, 0.37 [0.14–0.98]) [64]. These studies demonstrate how patient-specific DTs integrate cardiac imaging and electrophysiological data for personalized arrhythmia management.

Advances in sex-specific drug classification have emerged through comprehensive DT frameworks. Bai et al. developed an approach for sex-specific identification of Class III antiarrhythmic drugs by integrating in vitro measurements, in silico models, and machine learning [65]. Simulating drug effects on diverse cardiomyocyte populations (5,663 males and 6,184 females), they achieved high prediction accuracy (>89%) using sex-dependent Support Vector Machine algorithms. The study revealed gender differences attributed to lower IK1, INa, and Ito currents in females, highlighting the importance of sex-specific considerations in antiarrhythmic drug development.

### 2.2 Hemodynamic modeling and monitoring

A significant breakthrough in hemodynamic modeling came with the development of the LHMF [27]. This framework addresses three critical challenges: computational intractability of explicit methods, boundary conditions reflecting varying activity states, and accessibility of computing resources for clinical translation. LHMF achieved unprecedented accuracy with error rates between 0.0002%–0.004% when compared to explicit data of 750 heartbeats. The introduction of LHMFC (clustering approach) further optimized the framework by identifying hemodynamically similar heartbeats, enabling the simulation of 4.5 million heartbeats while requiring only 1,160 representative hemodynamic units. This advancement represents a significant step toward creating comprehensive cardiovascular DTs capable of long-term monitoring and prediction.

The integration of edge computing with DT technology has enabled real-time cardiac monitoring. The Cardio Twin architecture [28] represents a significant advancement in this domain, achieving 85.77% accuracy in classifying ECG segments with a precision of 95.53%. This system processes ECG signals in approximately 4.8 ms, demonstrating its capability for real-time analysis on edge devices. The framework’s success lies in its ability to provide continuous monitoring while maintaining data privacy and computational efficiency on edge devices.

### 2.3 Cardiovascular monitoring frameworks

The development of comprehensive monitoring frameworks has advanced through the integration of IoT sensors with DT technology. Iyer and Umadevi introduced the TwinCardio framework, a novel reference architecture for DT-enabled smart health monitoring specifically designed for cardiovascular disease detection and monitoring [66]. The framework incorporates TwinNet, a customized neural network designed for cardiovascular disease classification and prediction, enabling continuous data acquisition, simulation, and evaluation while maintaining security protocols. This human-in-the-loop approach facilitates integration between the patient’s physical world and the medical virtual world, addressing the alarming 30% increase in heart attack cases among individuals aged 25–44 between 2020 and 2023 through more precise and timely healthcare delivery.

### 2.4 Heart failure and surgery

Novel approaches to treating heart failure with preserved ejection fraction (HFpEF) have emerged through computational modeling [67]. The research investigated the mechanical relationship between left ventricular (LV) function and ascending aorta elasticity, demonstrating that releasing the LV apex from pericardial confinement could significantly improve cardiac function. The simulations revealed impressive improvements in various cardiac parameters: longitudinal strain increased from −4.8% to −8.2%, radial strain from 18.5% to 22.4%, and circumferential strain from −14.2% to −16.5%, while reducing average myofiber stress by 18%. These findings suggest promising new therapeutic approaches for HFpEF treatment.

Personalized digital heart technologies have transformed surgical planning, particularly in treating complex arrhythmias. The digital-heart identification of fat-based ablation targeting (DIFAT) technology [29] has revolutionized ventricular tachycardia (VT) ablation by incorporating infiltrating adipose tissue distribution in 3D models. This technology achieved significant reductions in ablation volumes (mean 1.87 ${\mathrm{cm}}^{3}$ vs 7.05 ${\mathrm{cm}}^{3}$ in clinical procedures) while maintaining high concordance with clinical outcomes. The system showed remarkable accuracy in predicting critical ablation sites, with 79% overlap between predicted targets and actual clinical ablation locations.

### 2.5 Population-scale cardiac digital twins

Large-scale population studies have leveraged DT technology to uncover fundamental insights into cardiac electrophysiology and its relationship with demographics and disease states. Qian et al. constructed an unprecedented cohort of 3,461 cardiac DTs from the UK Biobank plus 359 from an ischemic heart disease cohort using cardiac magnetic resonance images and electrocardiograms [68]. This population-scale approach revealed that sex-specific differences in QRS duration were fully explained by myocardial anatomy while myocardial conduction velocity remained similar across sexes but changed with age and obesity, indicating myocardial tissue remodeling. The study demonstrated that longer QTc intervals in obese females were attributed to larger delayed rectifier potassium conductance, providing mechanistic insights into sex-specific cardiac electrophysiology that would be impossible to obtain through traditional clinical studies alone.

## 3 Neural system digital twins

Significant advances have been made in understanding neurodegenerative disease progression through physics-based modeling. The integration of sophisticated imaging analysis with DT technology has revolutionized our understanding of neural system dynamics and pathologies, particularly in the context of protein misfolding diseases.

### 3.1 Neurodegenerative disease modeling

The Fisher-Kolmogorov equation with anisotropic diffusion successfully simulates misfolded protein spread across the brain in Alzheimer’s, Parkinson’s, and ALS [30]. This model replicates characteristic progression patterns from histological and biomarker data, supporting the prion-like hypothesis of protein propagation. Recent DT advances include Cen et al.’s approach for estimating disease-specific brain atrophy onset in multiple sclerosis, revealing progressive tissue loss begins 5-6 years before clinical symptoms [31]. DTs demonstrate promise for neurological condition detection and management. Abirami and Karthikeyan’s DT-based Healthcare System (DTHS) for Parkinson’s disease achieved 97.95% and 91.48% prediction accuracy using optimized fuzzy-based k-nearest neighbor classifiers [32]. Allen et al. developed a variational autoencoder-based DT model for ischemic stroke patients, forecasting clinical trajectories with simulated data virtually indistinguishable from real patient data [69]. These advances provide capabilities for personalized monitoring, earlier detection, and predictive modeling.

Breakthrough developments in early-stage neurodegenerative disease detection have emerged through integrating non-invasive recordings with DT technology. Amato et al. introduced the Digital Alzheimer’s Disease Diagnosis (DADD) model, deriving personalized AD biomarkers from electroencephalographic recordings [70]. In 124 participants with varying cognitive decline, digital biomarkers improved classification accuracy by 7% over standard EEG biomarkers, identified CSF biomarker-positive patients with 88% accuracy (vs. 58% for standard EEG), and predicted cognitive decline conversions with 87% accuracy. This approach is valuable for preclinical patients excluded from invasive procedures, potentially revolutionizing early-stage AD diagnosis through digital twins with non-invasive recordings.

### 3.2 Brain tumor analysis

Advanced imaging analysis has been revolutionized through the integration of DT technology with sophisticated ML approaches [33]. The combination of S3VM and improved AlexNet CNN achieved a feature recognition accuracy of 92.52%, with impressive segmentation metrics including a Jaccard coefficient of 79.55% and positive predictive value of 90.43%. This hybrid approach effectively addresses the challenges of processing large volumes of unlabeled brain imaging data while maintaining high computational efficiency. The system demonstrated superior performance in both binary-class and multi-class classification tasks, outperforming traditional ML methods by at least 2.76%.

Recent advances have expanded DT applications to treatment optimization for high-grade gliomas. Chaudhuri et al. developed a data-driven predictive DT methodology for optimal risk-aware clinical decision-making in radiotherapy [34]. Their approach integrates mechanistic modeling with Bayesian calibration to assimilate patient-specific magnetic resonance imaging data, creating personalized DTs that account for uncertainties in tumor biology. By solving multi-objective, risk-based optimization problems, the framework generates patient-specific optimal radiotherapy regimens that balance the competing clinical objectives of maximizing tumor control while minimizing toxicity. In their in silico cohort of 100 virtual patients, personalized treatments achieved either a median increase in tumor time to progression of approximately six days using the same total radiation dose as standard-of-care, or a significant median reduction in radiation dose by 16.7% (10 Gy) while maintaining equivalent tumor control. This framework demonstrates how DTs can enable anticipatory personalized treatment strategies that adapt to the heterogeneous response patterns observed in high-grade gliomas, potentially improving outcomes for patients who respond poorly to standardized approaches.

## 4 Respiratory system digital twins

### 4.1 Lung modeling

Computational lung modeling has emerged as a sophisticated tool for understanding respiratory mechanics and disease progression [35]. These models integrate multiple scales of analysis, from alveolar mechanics to whole-organ function, incorporating compartmental models, discrete micromechanical models, and continuum representations. The approach enables detailed simulation of lung biomechanics in both health and disease states, facilitating personalized treatment strategies and improved understanding of structure-function relationships. The models have demonstrated particular success in simulating various aspects of lung function, including airway resistance, alveolar deformation, and ventilation heterogeneity.

### 4.2 Respiratory monitoring

Recent innovations in respiratory monitoring have introduced novel statistical approaches for data enhancement [36]. Using ESP32 wi-fi channel state information sensors for unobtrusive monitoring, researchers achieved 92.3% accuracy in breathing rate estimation. The implementation of sophisticated ML techniques, particularly bagged trees ensemble algorithm, demonstrated impressive classification accuracies of 89.2% for binary-class and 83.7% for multi-class respiratory pattern recognition. This approach represents a significant advancement in non-invasive respiratory monitoring, combining high accuracy with practical applicability.

## 5 Metabolic and endocrine system digital twins

### 5.1 Diabetes management

Advanced DT technology has been developed for T1D management through complementary approaches. The Exercise Decision Support System (exDSS) [38] provides personalized recommendations for glucose management during exercise, demonstrating significant improvements in maintaining target glucose ranges (increasing from 80.2% to 92.3% during aerobic exercise). The system showed particular effectiveness in reducing hypoglycemia incidents from 15.1% to 5.1% during aerobic activities. Additionally, statistical virtual patient populations [71] have been created to evaluate artificial pancreas control algorithms, incorporating both single-hormone and dual-hormone models validated against clinical data from T1D patients. These models demonstrated robust performance across diverse patient characteristics and treatment scenarios.

A comprehensive framework for Type 2 Diabetes DTs [39] combines ML, multiomic data, and knowledge graphs to improve predictive accuracy and disease mechanism interpretation. The system leverages the Arivale dataset and SPOKE knowledge engine to enable personalized predictions of disease trajectories and treatment responses. The integration of proteomic and metabolomic data significantly enhanced the model’s predictive capabilities, particularly for key clinical markers like HbA1c and eGFR.

### 5.2 Insulin resistance

A multi-scale DT for adiposity-driven insulin resistance [40] integrates mechanistic models of glucose metabolism, body composition, and cellular insulin signaling. This comprehensive framework successfully simulates and predicts responses to dietary and pharmacological interventions, providing insights into the progression of insulin resistance and supporting personalized treatment strategies. The model effectively captures both short-term responses to meals and long-term adaptations to dietary changes.

### 5.3 Metabolic response

Advanced DT technology has been developed to predict metabolic responses to various dietary compositions and fasting schedules [72]. The system employs a mechanistic, multi-scale model encompassing both intracellular processes and organ-organ crosstalk, particularly focusing on liver function and protein metabolism. This model enables personalized predictions based on individual demographics and metabolic history, demonstrating strong validation against experimental data for both fed and fasted states.

### 5.4 Pediatric metabolism

Sex-specific, personalized metabolic whole-body models (WBMs) have been developed for newborns and infants [41]. These “infant-WBMs” integrate organ-specific growth, energy demands, and metabolic processes to provide insights into infant development and predict biomarkers for inherited metabolic diseases. The models demonstrate strong agreement with WHO growth standards and enable the simulation of dietary interventions, offering a valuable tool for understanding early-life metabolism and disease progression.

## 6 Hepatic system digital twins

Recent advances in hepatic DT technology have encompassed both regeneration modeling and predictive organ-on-chip simulations, representing significant progress in understanding liver function and drug metabolism.

### 6.1 Liver regeneration modeling

Liver regeneration modeling has seen significant advancement through the development of sophisticated DTs [73]. The mathematical mechanism-based model provides unprecedented insight into tissue microarchitecture and cellular interactions during regeneration. This approach enables the testing of various hypotheses about cell-cell interactions, quantifying regeneration dynamics through multiple parameters including dead cell area size, hepatocyte density, and spatial-temporal profiles of different cell types. The model successfully captures the complex interplay between various cell types, including Kupffer cells and hepatic stellate cells, in the regenerative process. Its ability to identify gaps in mechanistic relationships has proven valuable for guiding experimental design and understanding complex biological processes.

### 6.2 Real-time hepatic tissue simulation

Cutting-edge developments in real-time liver simulation have emerged through thermodynamics-informed graph neural networks. Tesán et al. presented a novel hybrid model that integrates the geometric bias of graph neural networks with physical constraints derived from metriplectic structure implementation [74]. This approach enables simulation of hepatic tissue with dissipative properties while maintaining remarkable generalization capability for previously unseen anatomies. The model predicts human liver responses to traction and compression loads in as little as 1.65 ms in optimal configurations, achieving relative position errors below 0.15% and stress tensor estimations with relative errors under 7%. The integration of thermodynamic principles ensures that the network satisfies physical laws during inference, making this approach particularly relevant for precision medicine and haptic rendering applications.

### 6.3 Organ-on-chip digital twins

Revolutionary advances in predictive organ-on-chip simulations have been achieved through the DigiLoCS platform. Aravindakshan et al. developed a comprehensive digital twin model of liver-on-chip systems that closely mimics human liver clearance functionality [75]. Using compartmental physiological models based on ordinary differential equations, the system estimates pharmacokinetic parameters related to on-chip liver clearance for drug development applications. The approach successfully predicted in vitro liver clearance for 32 drugs and demonstrated superior performance compared to conventional models in predicting intrinsic liver clearance. By establishing connections between hardware chip structure and advanced biological mapping, DigiLoCS enables differentiation between active biological processes (metabolism) and passive processes (permeability and partitioning), incorporating detailed compound-specific characteristics and hardware-specific data. This represents the largest cross-organ-on-chip platform investigation to date, systematically analyzing and predicting human clearance values to reduce time, cost, and patient burden in drug development.

## 7 Cancer and tumor digital twins

### 7.1 Cancer-specific models

Recent advances in cancer-specific DTs have demonstrated significant progress in predicting treatment responses and optimizing therapeutic strategies. [76] developed a systems-based DT approach for characterizing dose-response relationships in non-Hodgkin lymphoma, using quantitative systems pharmacology (QSP) to generate individualized virtual patients. This approach enabled the simulation of patient responses to varying dosing regimens, accounting for biological variability and competitive binding effects. In the domain of multi-organ DTs, significant progress has been made in cancer progression monitoring [77]. The implementation of natural language processing for analyzing structured radiology reports has enabled sophisticated tracking of metastatic disease across multiple organs, showing superior performance in detecting and monitoring cancer progression, particularly in the lungs, liver, and adrenal glands. The integration of consecutive report analysis has significantly improved detection accuracy, with models showing enhanced predictive power for identifying progression patterns across different organ systems.

### 7.2 Prostate cancer

The development of DTs for prostate cancer has focused on two main areas: prediction and pathology. An ML-based DT system was developed for predicting prostate cancer progression [42], achieving 96.25% accuracy in biochemical recurrence prediction using data from 404 patients. The system demonstrated particularly high performance when using all available clinical data, showing approximately a 4% improvement over traditional methods. Complementing this work, a critical evaluation of AI as a DT of pathologists [43] demonstrated comparable performance to human pathologists in detecting prostate cancer and estimating tumor volume, though noting challenges in grade discordance for prostatectomy specimens. The study achieved significant improvements in diagnosis efficiency while maintaining high accuracy levels comparable to human experts.

Advanced mechanistic modeling approaches have enhanced prostate cancer treatment through comprehensive DT frameworks. Stamatakos et al. developed a mechanistic multiscale model of clinical prostate cancer response to external radiation therapy as the core of a digital virtual twin [78]. This discrete entity and discrete event simulation approach incorporates patient-specific cancer biology in terms of radio resistance and individual patient preferences. The model demonstrated particular sensitivity to critical parameters including apoptosis rates of living stem and progenitor cells, the fraction of dormant cells reentering cell cycle, and the fraction of stem cells performing symmetric division. Following technical verification and sensitivity analysis, the model showed qualitative agreement with experimental and clinical knowledge, establishing the foundation for clinical validation and eventual certification for clinical translation as part of the envisaged OncoSimulator system.

### 7.3 Lung cancer

DT technology has demonstrated remarkable advancements in lung cancer management. The DT-GPT model integrates electronic health record data to forecast clinical variables for non-small cell lung cancer patients with high accuracy ($R^{2}$ of 0.98) and a 3.4% improvement in mean absolute error, effectively managing missing data while enabling zero-shot forecasting capabilities [79]. Complementing this trajectory modeling, the Lung-DT framework creates comprehensive digital representations of respiratory health by integrating IoT sensors with AI algorithms, specifically employing the YOLOv8 neural network to classify chest X-rays into five distinct categories with exceptional performance metrics (96.8% accuracy, 92% precision, 97% recall, and 94% F1-score) [37]. This framework enables real-time monitoring through continuous data acquisition, automated objective assessments of chest X-rays, and comprehensive correlation of multiple data streams, representing a significant advancement in thoracic healthcare delivery with potential benefits for early diagnosis, enhanced patient outcomes, reduced healthcare costs, and optimized resource allocation.

### 7.4 Head and neck cancer

DT technology has been applied to optimize treatment decisions for oropharyngeal squamous cell carcinoma (OPSCC) [44]. The system employs deep Q-learning with a patient-physician DT dyad, trained on data from 536 OPSCC patients, improving survival rates by 3.73% and reducing dysphagia rates by 0.75% while achieving an average prediction accuracy of 87% for treatment outcomes. Recent innovations by Männle et al. have extended DT applications to surgical planning and intraoperative guidance through an AI-directed framework that measures soft tissue shift during head and neck surgery [45]. Using a pig head cadaver model with 104 soft tissue resections, they created DTs of both removed tissue pieces and corresponding resection cavities using two different 3D scanners (HoloLens 2 and ArtecEva), demonstrating the ability to simulate and measure tissue deformation by inducing temperature changes, with resection cavities showing a volume increase of 3.1 μL or 9.09% upon heating. This approach addresses the previously unsolved problem of soft tissue shift detection during surgery, with potential applications in frozen section management and improved surgical precision, while validating that despite different point cloud densities between scanning devices, both provided comparable volume estimates suitable for clinical applications.

### 7.5 Pediatric oncology

The PRIMAGE project [46] introduces an innovative approach to pediatric cancer diagnosis and prognosis. This framework integrates imaging biomarkers, clinical data, and AI, specifically targeting neuroblastoma and diffuse intrinsic pontine glioma. The system achieved high accuracy in tumor segmentation with a Dice similarity coefficient of 0.997, significantly reducing radiologist workload by 93%. The framework’s success lies in its comprehensive approach to data integration and its ability to provide actionable insights for clinical decision-making.

### 7.6 Cancer metabolic monitoring

Patient-specific DTs have been developed for monitoring metabolic biomarkers in cancer patients [80]. Using Long Short-Term Memory (LSTM) recurrent neural networks, these models achieve relative errors below 10% for key biomarkers, enabling both retrospective analysis and short-term forecasting of patient health states. The system demonstrates successful transfer learning capabilities, allowing efficient adaptation to new patients while maintaining prediction accuracy. The framework’s ability to track multiple metabolic indices simultaneously makes it a valuable tool for comprehensive patient monitoring.

### 7.7 Tumor microenvironment

Understanding the mechanical and immunological aspects of tumor development has emerged as a crucial area in DT research. Loewke et al. [47] introduced a DT framework for Cellular Capsule Technology that investigates how mechanical stresses influence tumor growth and cellular dynamics, with their multiphase poro-mechanical model revealing critical insights into how capsule constraints affect tumor behavior and invasive phenotypes while successfully capturing complex interactions between tumor cells, interstitial fluid, and the extracellular matrix. Complementing this mechanical perspective, Rocha et al. [48] developed a multiscale mathematical model to study immune surveillance of micrometastases in epithelial tissues, generating over 100,000 virtual patient trajectories that recapitulated diverse clinical scenarios including uncontrolled growth, partial response, and complete immune response to tumor growth. Their work on cancer patient DTs (CPDTs) identified key parameters affecting simulated immunosurveillance. It also highlighted significant challenges in the field, including uncertainties in immune responses, unreliable patient stratification, and unpredictable personalized treatment outcomes. However, they demonstrated that patient-specific models can suggest strategies to increase the control of clinically undetectable micrometastases even without complete parameter certainty.

### 7.8 Geriatric and rare cancer applications

DT technology has expanded to specialized oncological populations. Heudel et al. developed a prognostic tool for geriatric breast cancer patients using AI and clinical-biological features [81]. Analyzing 793 women aged 70+ with HER2-negative early-stage breast cancer, their machine learning approach achieved AUC scores of 0.81, outperforming traditional models.

## 8 Cellular and molecular digital twins

Significant advances in metabolic modeling have emerged through DT applications, particularly in understanding complex biological systems and their interactions at the cellular level. These developments have enabled unprecedented insights into metabolic regulation and cellular behavior.

### 8.1 Metabolic modeling

Researchers developed microbial community-scale metabolic models for predicting personalized short-chain fatty acid production in the human gut, demonstrating strong correlations with clinical health markers and enabling the design of personalized interventions [82]. The models achieved significant predictive accuracy, with Pearson correlations of *r* = 0.62 for butyrate and *r* = 0.63 for propionate production. At the genome scale, [83] introduced a comprehensive method for modeling metabolism and gene product expression, successfully integrating biochemical reactions with transcription and translation processes. This approach provided a unified framework for understanding cellular physiology at multiple scales.

### 8.2 Drug development

DTs have significantly advanced our understanding of drug responses and resistance mechanisms. Authors in [54] demonstrated that human in silico drug trials achieve higher accuracy (89%) than traditional animal models (75%) in predicting cardiac pro-arrhythmic cardiotoxicity. Recent work by [55] has addressed the challenge of drug resistance in cancer treatment by developing a comprehensive framework that incorporates both irreversible and reversible resistance mechanisms, demonstrating improved patient outcomes through optimized treatment strategies.

### 8.3 Molecular communication

Advances in molecular communication and drug delivery have been significantly propelled by innovative modeling approaches. For example, Khoshfekr Rudsari et al. [84, 85] developed three-dimensional partial differential equation models to characterize both normal and anomalous diffusion of extracellular vesicles (EVs) within the extracellular matrix. By incorporating matrix-specific properties, these models accurately predicted EV biodistribution patterns and transport dynamics, particularly within cardiac tissue. Building on this foundation, recent DT-based studies have progressed from organ- and tissue-level simulations to modeling cellular and subcellular processes, particularly those governing cell–cell and cell–microenvironment interactions that modulate therapeutic response. Notably, Irajizad et al. [86] modeled mitochondrial fission by capturing the biophysical interactions between lipids and proteins, enabling detailed insights into the division mechanisms of mitochondria and identifying protein-binding interventions capable of disrupting this process. Similarly, DT models of endocytosis have elucidated how geometric instabilities emerging during the cell cycle drive vesicle formation, and how these instabilities can be regulated through targeted modulation of protein interactions [87].

### 8.4 Genetic dynamics

The role of genetic dynamics in treatment optimization has been explored through sophisticated modeling approaches. Authors in [88] investigated how genetic dynamics and single-cell heterogeneity impact personalized medicine strategies for cancer. Their work demonstrated that accounting for tumor evolution and genetic diversity can significantly improve treatment outcomes, particularly in the context of drug resistance development.

### 8.5 Perturbation prediction modeling

DTs of cells are virtual models designed to simulate the behavior and function of biological cells. A key subset of these DTs, commonly referred to as in silico prediction models, focuses on predicting cellular responses, often measured by gene expressions or protein expressions, to perturbations such as drugs, gene knockouts, or metabolic changes [89, 90]. These models play an important role in accelerating research in drug discovery and development by enabling in silico experiments [49], significantly reducing costs and saving valuable resources typically required for in vitro experiments [50].

**Figure 2:**
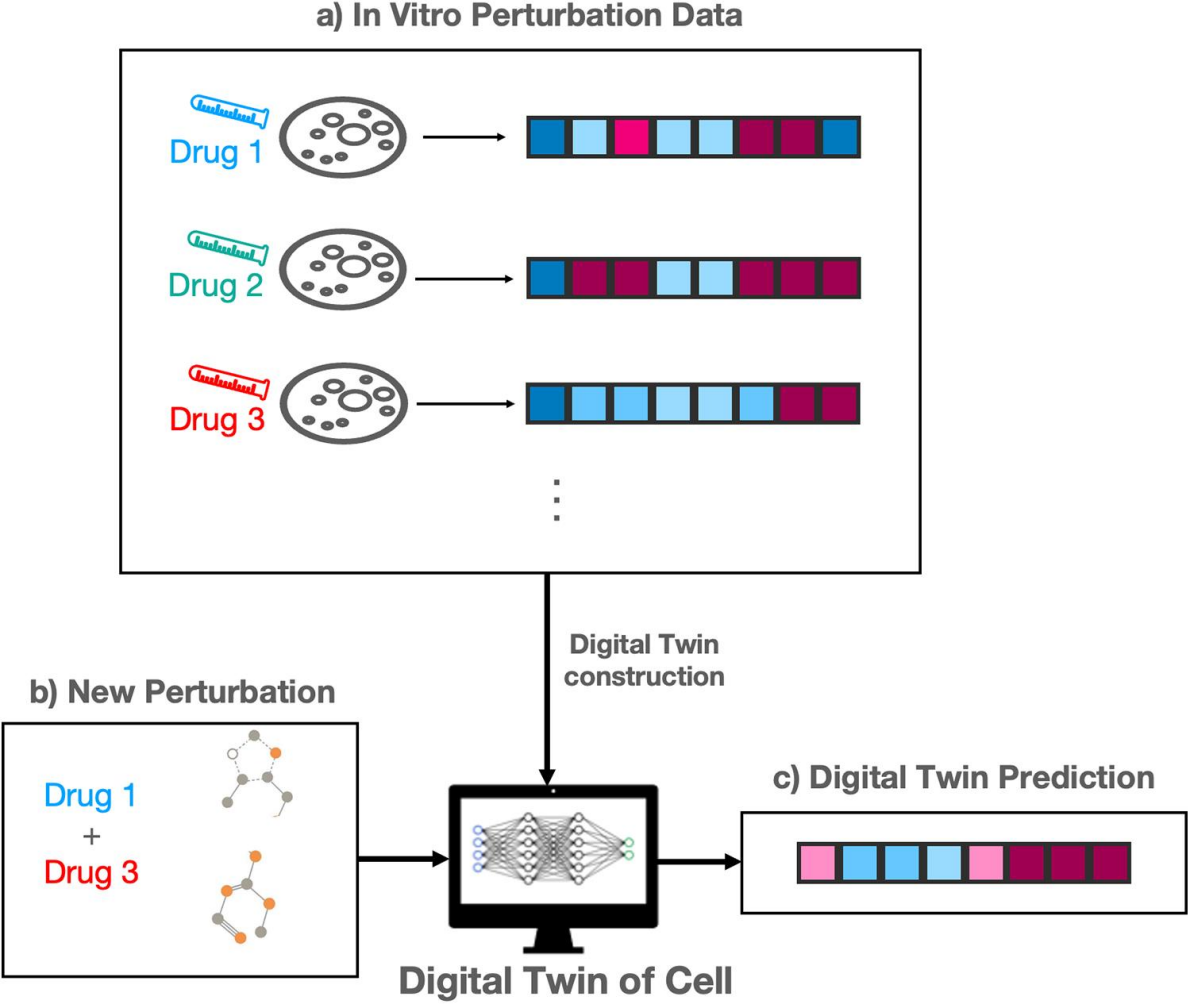
Outline of digital twin of cells for perturbation prediction modeling. **(a)** In vitro drug perturbation data provide baseline response profiles. **(b)** A new perturbation is introduced to the digital twin model. **(c)** The digital twin predicts the resulting profile response in silico.

Figure 2 outlines the construction and purpose of a cell DT for perturbation modeling. A large number of perturbations are performed in vitro (in a wet lab) and cellular responses measured (Figure 2a). These experiments are expensive to run, requiring substantial time and financial resources. The in vitro response data is used to construct a DT of the cell, also known as an in silico model. A researcher may then use the DT to predict cellular responses for some new perturbation, which has not be tested in vitro (Figure 2b). The twin predicts a response to this combination (Figure 2c), eliminating the need to run further expensive in vitro experiments.

## 9 Cross-system applications

### 9.1 Surgical and interventional systems

A novel DT-enabled IoMT system [60] has been developed for telemedical surgical simulation. The system integrates mixed reality, 5G cloud computing, and robust auxiliary classifier generative adversarial networks (rAC-GAN) to address complex surgical scenarios, achieving 92%–93% predictive accuracy. The framework demonstrated particular success in lung cancer cases complicated by pulmonary embolism, utilizing advanced deep learning techniques for real-time surgical simulation and decision support.

DT technology has been applied to magnetic medical microrobots [61], incorporating stochastic model predictive control enhanced by ML. The system demonstrates high precision in navigating complex biological environments, with applications in targeted drug delivery and minimally invasive surgeries. The integration of Kalman filtering and advanced control strategies enables robust performance even in noisy environments.

### 9.2 Treatment optimization

An in-silico model has been developed to analyze cytotoxic drug administration to solid tumors [91]. This DT incorporates multiple biological processes, including tumor growth, blood vessel development, and drug transport mechanisms. The model revealed significant insights into how drug binding affinity and blood vessel permeability influence treatment efficacy, with high-affinity drugs showing consistent effectiveness regardless of administration timing.

DTs have been developed to optimize transdermal fentanyl therapy for chronic pain management [92]. This individualized approach addresses patient variability in treatment response, leading to a 16% decrease in pain intensity and a 23 h increase in median pain-free time over 72 h periods. The model successfully integrated pharmacokinetics with patient-specific physiological parameters to enable precise dosing strategies.

### 9.3 Medical education

A DT application for critical care education [93] has been developed to simulate patient conditions and responses during the crucial first six hours of critical illness. The system demonstrates good usability with a median System Usability Scale score of 70, providing a realistic platform for training medical residents in complex clinical scenarios.

Patient-specific, three-dimensional mixed-reality anatomical models [62] have been developed for surgical training and intraoperative guidance. These models, derived from CT or MRI data, enable surgeons to interact with highly accurate representations of anatomy, improving understanding of complex structures and spatial relationships. The technology demonstrates significant advantages over traditional 3D-printed models, particularly in terms of cost-effectiveness and flexibility. The process requires 20–30 h for model creation but offers superior visualization and manipulation capabilities compared to conventional methods.

Mixed-reality anatomical models serve dual purposes in surgical education and training, enabling preoperative rehearsal and intraoperative guidance, while also facilitating remote teaching and collaboration through mixed-reality interfaces. The technology has shown particular value in complex surgical planning and training scenarios.

### 9.4 Healthcare infrastructure

An ISO/IEEE 11073-standardized framework [56] has been developed for health and well-being in smart cities. This system integrates compliant and non-compliant health devices through an X73 wrapper module, achieving classification accuracy up to 96.85% in activity tracking. The framework demonstrates significant potential for improving population health monitoring and management in urban environments.

Cloud-based design and additive manufacturing approaches have been developed for custom orthoses [94]. This DT application streamlines the design and production of personalized medical devices, such as custom ankle-foot orthoses, spinal braces, or wrist supports by integrating cloud computing and 3D printing technologies. The system has shown particular success in reducing production time and costs while maintaining high accuracy in custom device creation.

### 9.5 Clinical trials digital twins

Recent innovations in DT technology are transforming clinical trials through advanced modeling approaches that address critical challenges in patient recruitment, retention, and outcome prediction. Chandra et al. introduced ClinicalGAN, a generative adversarial network that creates patient DTs for clinical trial monitoring by enabling patient-level personalized generation using meta-data for conditional generation [16]. Validated on two Alzheimer’s clinical trial datasets, ClinicalGAN outperformed state-of-the-art approaches by 3%–4% across generation quality metrics and demonstrated 5%–10% improvement in mean absolute percentage error (MAPE) scores for patient drop-off prediction, offering powerful capabilities for proactive monitoring and improved retention.

These technological advancements align with broader industry trends, as Moingeon et al. highlight how virtual patients and DTs are increasingly being used to simulate in silico the efficacy and safety of drug candidates and medical devices [95]. Their work emphasizes the growing acceptance of digital evidence by regulators and how predictive AI-based models can support confirmatory trial design while accelerating drug and medical device development, pointing toward a future where computational models complement traditional clinical trials to improve efficiency, reduce costs, and enhance patient safety.

### 9.6 Clinical operations and infrastructure

DT ecosystems for clinical operations have transformed healthcare delivery through integrated frameworks that streamline workflows and enhance decision-making. These systems incorporate specialized twins for medical necessity evaluation, care navigation, and clinical history visualization, demonstrating particular success in oncology where they optimize treatment pathways and improve care coordination [57]. Standardized frameworks compliant with ISO/IEEE 11073 standards have enabled comprehensive health monitoring by integrating diverse health devices and data sources, achieving classification accuracy up to 96.85% while ensuring robust data collection and analysis cycles [56]. These implementations provide essential infrastructure for systematic health monitoring and clinical decision support.

Early Warning Systems leveraging DT technology have significantly improved patient safety outcomes, achieving a 60% reduction in code blue incidents through predictive analytics that identify early signs of patient deterioration [58]. This proactive approach has demonstrated substantial improvements in response times and patient outcomes through timely intervention strategies.

### 9.7 Chronic condition management

DT technology has been applied to chronic wound management [96], utilizing AI techniques to enhance clinical decision support and predict healing trajectories. The system employs generative adversarial networks for visual prediction, achieving approximately 74% accuracy in tissue distribution predictions. The framework demonstrates particular success in providing personalized treatment recommendations based on wound characteristics and healing patterns.

Advanced monitoring systems using DTs [80] enable tracking and forecasting of patient-specific metabolic indices. These systems achieve relative errors below 10% for key biomarkers and demonstrate successful transfer learning capabilities. The technology has shown significant potential in personalized medicine applications, particularly in monitoring and managing chronic metabolic conditions.

### 9.8 Large language model integration in digital twins

The integration of Large Language Models (LLMs) into digital twin architectures enables sophisticated cross-dataset associations and knowledge synthesis from heterogeneous data sources. Wang et al. developed TWIN-GPT, an LLM-based approach that establishes cross-dataset associations despite limited data availability, generating personalized DTs that enhance clinical trial outcome prediction while producing high-fidelity trial data in data-scarce situations [59]. This capability addresses critical limitations in precision medicine where rare conditions often lack sufficient training data, enabling transfer learning across diverse clinical contexts to inform patient-specific predictions even when direct observational data remain sparse.

At the cellular level, LLM applications have revolutionized perturbation prediction and single-cell analysis. ScFoundation features read-depth-aware pre-training for handling sparsity in single-cell RNA sequencing data [97], while scGPT treats gene expression profiles as “sentences” and genes as “tokens,” achieving state-of-the-art performance in predicting genetic perturbation responses and multi-omic integration [53]. For rare malignancies, Lammert et al. developed LLM-enabled digital twins for metastatic uterine carcinosarcoma by integrating clinical data from 21 patients with 655 publications, identifying treatment options missed by traditional analysis and facilitating a shift from organ-based to biology-based tumor definitions [98].

## 10 Methods in building digital twins

### 10.1 Data collection and integration

DT development relies on diverse data sources including sensor-based monitoring, medical imaging, and EHR, enhanced by real-time data streaming for dynamic updates and predictive capabilities. IoT devices enable early detection of potential problems through continuous physiological monitoring [99].

Multiple sensor types collect real-time patient data: biometric sensors measure vital signs (heart rate, blood pressure, temperature), movement sensors track activity and gait patterns, electrophysiological sensors monitor electrical activity like Electroencephalography (EEG) and electromyography (EMG), and chemical sensors detect biomarkers such as glucose levels [100]. Medical imaging scans including magnetic resonance imaging (MRI), computed tomography (CT), and ultrasound provide detailed anatomical information, requiring sophisticated image processing algorithms, segmentation techniques, and ML models for integration into patient-specific computational models [101].

EHR integration presents both opportunities and challenges, providing valuable repositories of patient histories, laboratory results, medication records, and clinical notes, while facing issues of data heterogeneity, interoperability, and privacy concerns. Standardized protocols such as Fast Healthcare Interoperability Resources (FHIR) [102] and Health Level Seven (HL7) enable data integration across healthcare systems [103]. Real-time data streaming employs edge computing, cloud-based analytics, and federated learning for rapid processing and synchronization, with frameworks including Apache Kafka and Message Queuing Telemetry Transport (MQTT) protocols enabling efficient handling of high-velocity medical data streams [104].

This integrated approach forms the foundation of healthcare DT technology, with continued evolution in AI, data interoperability, and secure data-sharing frameworks driving unprecedented precision in personalized medicine and predictive healthcare.

### 10.2 Perturbation datasets for perturbation cell prediction modeling

Recently, researchers have generated datasets suitable for training in silico prediction models. The LINCS platform [105] provides 1,000 measured gene expression profiles for 71 cell lines and over 25,000 perturbations, including small molecule compounds, gene knockdowns or overexpressions, and biologics. Approximately 10% of the possible 1.75 million perturbation experiments were conducted, leaving significant room for predictive models to estimate cellular responses for the vast number of untested cell line-perturbation combinations.

A 2023 Kaggle competition [106] introduced another perturbation dataset suitable for training predictive models. The dataset originates from a novel single-cell perturbation experiment conducted on peripheral blood mononuclear cells (PBMCs). It includes 18,211 gene expression profiles following treatment with 144 compounds across six PBMC cell types. The experiments were performed on PBMCs from three healthy donors, allowing downstream analysis to discover population-level biological findings.

Another valuable source of datasets is scPerturb [49], which comprises a collection of 44 publicly available single-cell perturbation-response datasets with molecular readouts, including transcriptomics, proteomics, and epigenomics. These datasets provide researchers with the flexibility to develop and train various in silico prediction models, contributing valuable resources to advance this field.

### 10.3 Modeling approaches

#### 10.3.1 Perturbation cell prediction models

##### 10.3.1.1 Dynamic models

In silico prediction models using differential equations capture biological system dynamics, particularly suitable for modeling gene-regulatory networks (GRNs), metabolic pathways, and time-dependent processes. This approach enhances mechanistic interpretability for analyzing feedback loops and regulatory interactions, incorporates nonlinearity characteristic of biological systems, and enables predictive studies through parameter adjustments.

Bicycle [51] infers causal relationships in cyclic GRNs using stochastic differential equations, predicting gene expression through latent space modeling, intervention-specific parameters, and steady-state dynamics. Cellbox [52] models cellular systems with nonlinear ordinary differential equations, where parameters represent interaction strength and direction between cellular components, linking molecular to phenotypic changes and generalizing to unseen perturbations. Cellbox was augmented with adjoint sensitivity optimization [107], applying adjoint methods for efficient parameter optimization with backward optimization over full system trajectories using high-order ODE solvers. Other works include a graph variational Bayesian causal inference framework [108] and sc-OTGM [109], which predicts cell responses by solving optimal mass transport on Gaussian mixture manifolds.

##### 10.3.1.2 Generative models

In silico prediction models leverage deep learning and LLM approaches to model cellular behavior and predict perturbation responses. While less biologically interpretable than dynamic models, their complex network structures represent cellular signaling pathways, offering alternative perspectives on cell dynamics.

Lotfollahi et al. introduced variational autoencoder-based models including Single-cell Generative Network (scGen) [110], Transfer Variational Autoencoder (trVAE) [111], and Compositional Perturbation Autoencoder (CPA) [112]. These models assume scRNA-seq data reside on low-dimensional manifolds, using VAEs to map high-dimensional expression data into latent space for interpolating between cell states and capturing perturbation-specific and cell type-specific patterns.

Graph Enhanced Gene Activation And Repression Simulator (GEARS) [113] integrates biological knowledge into graph neural network (GNN) architecture using gene coexpression and perturbation graphs. It predicts perturbed gene expression by mapping gene embeddings and cross-gene effects to transcriptional space.

##### 10.3.1.3 Alternative modeling approaches

Some alternative models use novel approach to predict cellular responses to perturbations but do not exactly model cells. SI-A [50] is one of the examples that uses the synthetic intervention framework to predict the target cellular responses by building a synthetic version from the donor observations. The model assumes a latent factor model and a linear causal DAG of latent factors and gene interactions. Under these assumptions, the model proves the consistency of the synthetic intervention estimator, providing a robust framework for causal inference in cellular perturbation experiments.

#### 10.3.2 Physics-based modeling

Physics-based models describe biological systems using first principle equations, simulating key biological factors and their interactions to understand observed behaviors. These models employ discrete methods (where components are unique entities with defined states, locations, and behavioral rules) or continuum descriptions using spatial/spatiotemporal equations (ordinary or partial differential equations) representing average behaviors across regions or time.

Discrete methods represent individual amino acids for *de novo* protein modeling, individual cells within tissues, organs within individuals, or whole patients for infectious disease spread modeling [114]. Continuum methods study fluid flow using Navier-Stokes equations in cardiovascular systems [27] or Fick’s law-based molecular diffusion within tissues. Discrete and continuum components can be hybridized for more complete biophysical system descriptions.

Physics-based models excel in applications with limited experimental or clinical data, requiring only single-patient measurements. They identify underlying mechanisms responsible for behaviors of interest and enable in silico study of how biophysical factor changes affect system behavior.

##### 10.3.2.1 Continuum models

Continuum models describe changes in quantities of interests, as well as their feedback and interactions, using ordinary or partial differential equations (usually functions of time or space and time, respectively). These models are especially useful to study how molecules move among multiple systems or organs in the body, and they have been successfully applied to diverse problems, such as hemodynamic flow [27], neurodegenerative diseases (Section 4.2) [30], insulin resistance [40], and even cancer treatment with checkpoint inhibitor immunotherapy based only on clinically available data [115]. Table 2 presents common equations used in continuum modeling approaches.

**Table 2:** Common equations in continuum models for biological systems.

Model type	Governing equation
Compartmental ODE model [154, 155]	$\frac{dC_{i}}{dt}=\sum_{\;j}k_{\;ji}C_{j}-\sum_{\;j}k_{ij}C_{i}+S_{i}-D_{i}C_{i}$
Reaction-diffusion equation [156, 157]	$\frac{dC}{dt}=\nabla \cdot (D\nabla C)+R(C,t)$
Navier-Stokes equations [158, 159]	$\rho \left(\frac{\partial \vec{v}}{\partial t}+\vec{v}\cdot \nabla \vec{v}\right)=-\nabla p+\mu \nabla^{2}\vec{v}+\vec{\;f}$
	$\nabla \cdot \vec{v}=0$
Fick’s First law (diffusion) [160, 161]	J=−D∇C
Lotka–Volterra predator–prey model [162]	$\frac{dx}{dt}=\alpha x-\beta xy$
	$\frac{dy}{dt}=-\gamma y+\delta xy$
Linear quadratic model [163]	$S=\exp(-\alpha D-\beta D^{2})$

$C_{i}$ represents the concentration of a molecule in compartment i, $k_{ij}$ represents the transfer rate between compartments, $S_{i}$ represents sources, $D_{i}$ represents decay rates, D is the diffusion coefficient, R(C) represents reaction terms, $\vec{v}$ is the velocity field, p is pressure, ρ is density, μ is viscosity, $\vec{\;f}$ represents external forces, and J is the diffusion flux. Note that the reaction-diffusion equation simplifies to Fick’s Second Law of diffusion if R(C,t)=0. In the Lokta-Volterra predator-prey model, x and y are population densites of prey and predator species over time, α and β are per-captia growth (birth) rates and death rates due to predation in the prey population, and λ and δ are death rates and rate of population increase facilitated by the prey population. The linear quadratic model describes the portion of surviving cells (S) after radiation dose (D) as a function of fitted parameters α and β that correspond to linear and quadratic response patterns.

These examples used systems of ordinary differential equations to study the rates at which molecules of interest enter and leave connected systems across the body and interact with other molecules based on system specific properties (such as molecular flux, diffusion, consumption, or reaction rates) to understand bidirectional interactions between system and molecular properties and their roles in homeostasis or disease development and progression. If the spatial distribution of molecules is relevant to the problem being studied, partial differential equations may be used, as shown in Table 2. Examples of DT applications include molecular communication via extracellular vesicles [84, 85].

The same methods are also commonly applied to systemic drug delivery to calculate how much drug is delivered to the target, limiting factors, and estimate the dosage required to achieve a therapeutic effect. These are referred to as pharmacokinetic (PK; the process of drug absorption, delivery, elimination) or pharmacodynamic (PD; quantify drug mechanisms of action and effects), and can be hybridized into PK/PD models [116]. Table 3 presents examples of some of the fundamental equations commonly used in these approaches.

**Table 3:** Pharmacokinetic/Pharmacodynamic (PK/PD) model equations.

Model type	Governing equation
Basic pharmacokinetic model [164, 165]	$\frac{dC_{p}}{dt}=k_{a}D_{\text{abs}}-k_{e}C_{p}-\sum_{i}k_{i}C_{p}$
	$\frac{dD_{\text{abs}}}{dt}=-k_{a}D_{\text{abs}}$
Hill equation (pharmacodynamic) [166, 167]	$E=E_{\text{max}}\frac{C^{n}}{C^{n}+{\text{EC}}_{50}^{n}}$

$C_{p}$ is the plasma concentration, $D_{\text{abs}}$ is the amount of drug at the absorption site, $k_{a}$ is the absorption rate constant, $k_{e}$ is the elimination rate constant, $k_{i}$ are rate constants for distribution to various tissues, E is the effect, $E_{\text{max}}$ is the maximum effect, C is the drug concentration, ${\text{EC}}_{50}$ is the concentration producing 50% of the maximum effect, and n is the Hill coefficient.

These models, as seen in Table 3, have been especially fruitful in studying cancer therapeutic delivery, including chemotherapy [117], checkpoint inhibitor immunotherapy [118], and nanoparticle-encapsulated drug delivery [119], and they can even be hybridized with discrete agent-based methods [116] to study how the drug affects tumors at the individual cell level. Importantly, because these models quantify the mechanistic links between the disease, the individual patient’s characteristics, and simulated outcomes, they allow for simulation of a wide range of potential treatment outcomes for DT-based treatment optimization (see also Section 11.1.1).

##### 10.3.2.2 Discrete models

Discrete models employ unique representations of entities to be studied, which may range from individual people to molecules or atoms. By imposing a set of rules and states on each agent, models can study how these rules and associated outcomes and interactions between agents at a discrete scale sum to outcomes at population scales. Commonly, discrete entities are referred to as agents, and these models are known as agent-based models (ABMs). At the human scale, these are often used to study transmission of infectious disease or effects of health behaviors on populations [120]. Although these examples are not necessarily physics based, many agent-based models depend on physics. Table 4 presents the fundamental equations used in discrete modeling approaches. However, these models are often complex and highly customized to the application of study; widely-applicable equations are less common. Discrete models may be programmed according to sets of discrete Boolean rules based on simple equations (e.g., logistics of Heaviside functions for binary decisions) or even based on randomized outcomes, avoiding the need for governing equations altogether.

**Table 4:** Common equations in discrete Models for biological systems.

Model type	Governing equation
Molecular dynamics force field [168, 169]	$E_{\text{total}}=\sum_{\text{bonds}}K_{r}(r-r_{\text{eq}}{)}^{2}+\sum_{\text{angles}}K_{\theta }(\theta -\theta_{\text{eq}}{)}^{2}$
	$\;+\sum_{\text{dihedrals}}\frac{V_{n}}{2}[1+\cos(n\phi -\gamma )]$
	$\;+\sum_{i<j}\left[\frac{A_{ij}}{r_{ij}^{12}}-\frac{B_{ij}}{r_{ij}^{6}}+\frac{q_{i}q_{j}}{4\pi \varepsilon_{0}r_{ij}}\right]$
Cell-Cell interaction forces [170, 171]	${\vec{F}}_{ij}=k_{\text{rep}}\left(\frac{1}{r_{ij}^{n}}-\frac{1}{r_{0}^{n}}\right){\vec{r}}_{ij}+k_{\text{adh}}e^{-\frac{(r_{ij}-r_{\text{adh}}{)}^{2}}{2\sigma^{2}}}{\vec{r}}_{ij}$

In the force field equation, the first term represents bond stretching with force constant $K_{r}$ and equilibrium distance $r_{\text{eq}}$, the second term represents angle bending with force constant $K_{\theta }$ and equilibrium angle $\theta_{\text{eq}}$, the third term represents torsional rotation with barrier height $V_{n}$, periodicity n, and phase γ, and the fourth term represents non-bonded interactions (van der Waals and electrostatic) with parameters $A_{ij}$, $B_{ij}$ for van der Waals interactions and charges $q_{i}$, $q_{j}$. In the cell-cell interaction equation, ${\vec{F}}_{ij}$ is the force between cells i and j, $r_{ij}$ is their distance, $k_{\text{rep}}$ and $k_{\text{adh}}$ are repulsion and adhesion coefficients, $r_{0}$ is the equilibrium distance, and $r_{\text{adh}}$ and σ control the range of adhesion.

For example, models using discrete representations of atoms or small molecules are often employed to study molecular or protein conformation based on force field variants obtained from molecular dynamics simulations, such as AMBER [121] or CHARMM [122], as illustrated in Table 4. By representing covalent bonds as springs, including weak forces such as hydrogen bonds and van der Walls forces, and enforcing penalties to prevent overlap of atoms within the simulations, these simulations are able to generate and provide reliable protein conformation predictions. The strengths of these approaches are now being combined with deep learning methods such as AlphaFold2 [123] to further improve results.

Individual cells are also often represented as agents (sometimes referred to as cellular automata), which commonly interact with each other based on physical rules such as repulsion, adhesion, and deformation that are solved based on physics-engine algorithms, as shown in the second row of Table 4. Cell-scale ABMs have been applied to in silico study of organogenesis, for example the mammary gland [124], organ repair after damage (see Section 10.3.2.3) [73] and tumorigenesis. Powerful open-source tools like PhysiCell [125] are now enabling faster advancement in this technically challenging field by supporting the development of complex cell-scale ABMs.Commonly, cells within an ABM are combined with a continuum scale, allowing for chemical signaling among cells and explicit feedback between cells and their microenvironment.

##### 10.3.2.3 Hybrid discrete-continuum models

Hybrid models leverage the strengths of discrete and continuum models to generate more complete descriptions of biological systems. For example, users may choose to describe the cells within a tissue as discrete agents, while representing small molecule (oxygen, glucose, drug, etc.) movement through the tissue using continuum descriptions (e.g., Fick’s law) because the complexity of modeling each molecule is computationally prohibitive or not advantageous to the problem being studied. Table 5 presents examples of fundamental equations used in hybrid modeling approaches. The methods discussed in Sections 10.3.2.1, 10.3.2.2 can also be combined in clever and often complex ways to generate hybrid models that combine governing equations and Boolean decision making, enabling simulations of highly diverse phenomena across multiple scales.

**Table 5:** Hybrid discrete-continuum model equations.

Component	Governing equation
Molecular diffusion [172, 173]	$\frac{\partial C}{\partial t}=D\nabla^{2}C-\sum_{i}U_{i}({\vec{x}}_{i},C)+S(\vec{x},t)$
Cell movement [174, 175]	$\frac{d{\vec{x}}_{i}}{dt}={\vec{v}}_{i}(C,{\vec{F}}_{i})$
Cell force balance [125, 171]	$\frac{d{\vec{F}}_{i}}{dt}=\sum_{j}{\vec{F}}_{ij}+{\vec{F}}_{\text{ext}}$

C is the concentration field of a signaling molecule, D is the diffusion coefficient, $U_{i}$ represents uptake by cell i at position ${\vec{x}}_{i}$, S represents sources, ${\vec{v}}_{i}$ is the velocity of cell i which depends on the concentration field and forces, ${\vec{F}}_{i}$ is the total force on cell i, ${\vec{F}}_{ij}$ is the force between cells i and j, and ${\vec{F}}_{\text{ext}}$ represents external forces acting on the cell.

Even so, the complexity and computational costs for hybrid models, as represented in Table 5, commonly limits them to DT descriptions or tissues or organs (i.e, subsystems) within a patient. Examples include studying liver regeneration after drug-induced damage (Section 8) [73], how endocrine and paracrine signaling influence organ development on a cellular level [124], and how phenotypic hierarchies and hormonal signaling influence the development and progression of hormone-dependent tumors such as breast cancer [126]. This approach allows for complex, multiscale study of detailed behaviors that are often not observable in vivo or in vitro, revealing new insights into disease behaviors, promising treatment strategies, and new therapeutic targets.

## 11 Statistical and artificial intelligence modeling

Statistical and AI modeling techniques have become indispensable in constructing and refining DTs within healthcare. These methods enable predictive analytics, patient-specific outcome forecasting, and support for medical decision-making across a wide spectrum of diseases and organ systems. By leveraging large datasets—ranging from EHRs and omics profiles to sensor signals—researchers can develop sophisticated statistical and AI algorithms that mimic biological and clinical processes in silico. This section outlines the core statistical and AI-based modeling strategies commonly used to build healthcare DTs, highlighting both foundational and emerging methods.

### 11.1 Classical statistical approaches

Classical statistical models remain popular due to their interpretability and ease of implementation. Although few DT frameworks rely exclusively on traditional statistical methods, they are frequently employed as baseline or evaluation approaches to validate prediction using learned feature representation against ground truth data, thereby helping to benchmark the performance of more advanced modeling.

#### 11.1.1 Regression models

Traditional regression models remain integral in building DTs, offering interpretable insights into the relationships between predictive features and outcomes [25, 27, 39, 42]. Linear regression is used to relate predictors (e.g., demographics, clinical markers) to a continuous response variable (e.g., blood pressure, metabolic indices), and logistic regression is used for modeling dichotomous outcomes (e.g., disease vs. no disease). Generalized linear models can be used for modeling outcome following other distributions such as Poisson or negative binomial distribution (e.g., modeling counts of hospital admission in hospital operations).

#### 11.1.2 Survival analysis models

Survival analysis, such as the Cox proportional hazards model, estimates hazard functions for time-to-event data while handling censored observations [127]. In DTs focused on oncology or chronic diseases, survival analysis might be used to predict the time until tumor recurrence or a major adverse cardiac event. By continuously updating patient-specific factors (e.g., changes in lab results, new symptoms), the DT refines risk estimates over time.

### 11.2 Machine learning and deep learning

While classical statistical methods offer interpretability and a well-established theoretical foundation, they often rely on linear assumptions and manually selected features that may not fully capture the complexities of large-scale healthcare data. In contrast, ML [128] and DL [129] techniques excel at identifying non-linear patterns, extracting high-dimensional representations, and integrating a wider range of data sources(from clinical notes and sensor readings to multi-omics profiles and imaging) without requiring extensive feature engineering or predefined functional forms [130].

#### 11.2.1 Tree-based ensemble methods

Tree-based ensemble methods, such as Random Forests, Gradient Boosting Machines, and Extreme Gradient Boosting (XGBoost), have gained prominence in DT applications due to their robustness, interpretability, and strong predictive performance across diverse healthcare datasets [69, 131–135].

Tree-based ensemble methods work by training multiple decision trees and then combining the outputs to generate a final prediction [136, 137]. In Random Forest, each tree is built on a random subset of the training data and a random subset of features, thereby capturing diverse patterns and reducing overfitting [138]. Gradient Boosting methods, on the other hand, build trees iteratively, with each tree focusing on correcting the prediction errors of the previous one [139]. By combining the results of several weak learners (individual trees) into a single “ensemble,” these approaches often outperform individual decision trees. The final output, typically an average (for regression) or a majority vote (for classification), reflects the aggregated knowledge of all the trees. This design allows tree-based ensembles to handle noisy, high-dimensional data and naturally rank the importance of input features, making them highly valuable for DT applications, where data integration and interpretability are crucial.

#### 11.2.2 Neural networks and control systems

Neural networks have become a central component in DT applications thanks to their capacity for learning rich, non-linear representations from high-dimensional healthcare data [140–142]. Neural networks can automatically discover complex patterns at different levels through multi-layer architecture and back-propagation, leading to latent representations that capture underlying physiological or pathological states.

Different neural network architectures specialize in various tasks in DT systems. For example, Convolutional Neural Networks are highly effective for processing medical images and segmenting disease-relevant regions [143], while Recurrent Neural Networks and transformers excel at modeling time-series data (e.g., electrocardiograms or patient trajectories) [14, 144, 145].

While neural networks demonstrate remarkable predictive power, they can be prone to overfitting and may require large-scale, well-annotated datasets. Techniques such as data augmentation, regularization, and model distillation can help address these limitations. In parallel, emerging research integrates domain knowledge (e.g., known physiological constraints) directly into model architectures or training objectives, yielding models that are both high-performing and more interpretable.

Advanced control methodologies for medical DTs utilize neural network-based approaches. Böttcher et al. developed dynamics-informed neural-network controllers for agent-based biomedical models [146], addressing challenges in multi-scale, stochastic biological systems like immunity. Their methods demonstrated effectiveness with uncertainty quantification, advancing control theory application to complex biomedical systems.

#### 11.2.3 Synthetic classification approach

TabPFN (Tabular Prior-data Fitted Network) is a transformer-based foundation model for tabular data across biomedicine, economics, climate science, and materials research [147]. Unlike traditional ML requiring dataset-specific training, TabPFN learns prediction algorithms through meta-learning on millions of synthetic tasks. It delivers high-accuracy predictions on new datasets (up to 10,000 samples) in seconds via single forward passes, outperforming gradient-boosted decision trees. Beyond classification, TabPFN supports fine-tuning, data generation, density estimation, and transferable embeddings, positioning it as a transformative tool for accelerating scientific discovery across disciplines.

## 12 Discussion

### 12.1 Current limitations

The implementation of DTs in healthcare faces several critical challenges that must be addressed to realize their full potential. These limitations span technical, clinical, and regulatory domains.

#### 12.1.1 Data quality and availability

The development of accurate healthcare DTs requires comprehensive, high-quality patient data that is often fragmented across disparate systems [102, 103]. Patient-specific models demand extensive longitudinal datasets encompassing multiple physiological parameters, all of which remain difficult to collect and integrate [99–101]. This challenge is particularly pronounced for rare diseases and underrepresented populations, where limited data availability may compromise model generalizability. Furthermore, issues with data standardization, completeness, and interoperability continue to hinder seamless integration of information from various sources, including EHRs, imaging systems, and wearable sensors [102, 104].

#### 12.1.2 Computational constraints

Many sophisticated DT implementations, particularly those involving complex physiological systems or high-resolution imaging, impose significant computational demands [25, 27]. Physics-based models employing detailed finite element analysis or agent-based simulations often require specialized high-performance computing infrastructure that may not be readily available in clinical settings [35, 73]. The computational intensity of maintaining updated DT models in real-time presents a substantial barrier to widespread adoption, particularly for resource-constrained healthcare environments [28, 58]. These computational limitations often necessitate trade-offs between model complexity, accuracy, and practical utility in clinical workflows.

#### 12.1.3 Implementation complexity

Integrating digital twins into existing clinical workflows remains challenging due to technical, operational, and human factors [57]. Healthcare systems frequently operate with legacy technologies that may not readily accommodate the sophisticated infrastructure required for DT implementations [56, 58]. The complexity of deployment extends beyond technical considerations to include staff training, workflow redesign, and establishing protocols for interpretation and action based on DT insights [93]. This multifaceted implementation challenge requires coordinated efforts across technical teams, clinical staff, and healthcare administrators.

Provider adoption represents a particularly significant barrier to health DT (HDT) implementation, as it may be hindered by the inherent opacity of these sophisticated systems [148]. Amid ongoing frustrations with existing technologies like EHR and common concerns about bias in AI models, transparency and education regarding HDTs become crucial for facilitating both provider and patient buy-in, potentially during the informed consent process [148]. The economic framework for HDT adoption adds another layer of complexity, as implementation success is largely determined by payment considerations that may require novel financing approaches beyond traditional fee-for-service models, such as value-based payment schemes that reward improved patient outcomes without resulting in overutilization [148].

#### 12.1.4 Validation challenges

Establishing the validity and reliability of DT models in healthcare presents unique difficulties given the stakes involved in clinical decision-making [54, 63, 64]. Traditional validation approaches often rely on historical data, which may not adequately represent future patient populations or novel clinical scenarios [31, 49]. Prospective validation studies are resource-intensive and time-consuming, particularly for chronic conditions requiring long-term follow-up [40, 72]. Furthermore, validating DTs against gold standard measures can be problematic when such standards themselves have limitations or when the DT aims to provide insights beyond what conventional approaches can measure [33, 43]. The lack of standardized validation frameworks specific to healthcare DTs further complicates this challenge.

#### 12.1.5 Privacy and security concerns

The comprehensive nature of DTs raises significant privacy and security considerations, as these models integrate sensitive personal health information from multiple sources [28, 102]. Ensuring robust data protection while maintaining model accessibility for clinical use requires sophisticated technical safeguards and governance frameworks [103, 104]. The tension between data utility and privacy protection represents an ongoing challenge, particularly as DTs become more integrated into care delivery systems [2, 7].

#### 12.1.6 Digital equity

Healthcare DT implementation raises significant concerns about digital equity and bias that must be proactively addressed. Key challenges include ensuring access to necessary technology infrastructure in underserved and rural areas, developing diverse datasets to mitigate biases [as marginalized communities are often underrepresented in health data [149]], and addressing the digital skills gap among providers and patients. Without inclusive design and transparent governance, DT implementations risk exacerbating healthcare disparities rather than addressing them [149]. Establishing ethical guidelines, ensuring transparency in data usage, and fostering meaningful community engagement are essential to prevent the technology from primarily benefiting privileged populations while leaving vulnerable groups behind.

#### 12.1.7 Regulatory pathways and clinical translation

The clinical translation of healthcare DTs requires navigating evolving regulatory frameworks for validation and certification. The U.S. Food and Drug Administration (FDA) has established pathways for *in silico* clinical trials through its Medical Device Development Tools (MDDT) program, which provides qualification processes for computational models in regulatory submissions [150]. The European Medicines Agency (EMA) similarly supports modeling and simulation, including virtual patient populations, to supplement traditional clinical trial data [151]. International standards, notably the ASME V&V 40 standard, provide verification and validation frameworks specifically for computational models in medicine [152]. Despite this progress, significant challenges remain in establishing unified regulatory criteria for DT certification, particularly for real-time adaptive models and AI-integrated systems [153].

### 12.2 Future opportunities

Despite these challenges, DTs present transformative opportunities for advancing healthcare delivery, research, and patient outcomes.

#### 12.2.1 Enhanced personalization

The evolution of DTs promises unprecedented levels of treatment personalization across various medical domains [4, 11, 12]. Future DTs will likely integrate increasingly diverse data types, including multi-omics profiles, environmental exposures, behavioral factors, and social determinants of health, enabling truly holistic patient representations [13, 39]. This comprehensive approach will support precision interventions that account for individual variability at multiple levels, from molecular pathways to lifestyle factors [14, 15]. The potential for DTs to simulate patient-specific responses to different treatment options before actual implementation represents a paradigm shift in therapeutic decision-making, particularly for complex conditions with heterogeneous manifestations [26, 34, 64].

#### 12.2.2 Improved prediction accuracy

Advances in AI and computational methods will continue to enhance the predictive capabilities of DTs [37, 42, 79]. The integration of sophisticated ML architectures with mechanistic models promises to combine the pattern recognition strengths of AI with the biological plausibility of physics-based approaches [30, 51, 52]. Emerging techniques in explainable AI and uncertainty quantification will improve both the accuracy and interpretability of predictions, addressing critical requirements for clinical adoption [53, 112, 113]. These advancements will be particularly valuable for forecasting disease progression, treatment responses, and potential complications, enabling more proactive and preventive care approaches [38, 44, 80].

#### 12.2.3 Expanded application areas

While cardiovascular, neurological, and oncological applications currently dominate the DT landscape, future implementations will likely extend to additional medical domains and cross-system applications [29, 46, 77]. Promising areas include autoimmune disorders, psychiatric conditions, pediatric development, and geriatric syndromes [32, 41, 69]. Beyond individual patient care, DTs hold potential for population health management, health system optimization, and public health emergency response [56–58]. The development of DT ecosystems that model interactions between multiple physiological systems will enable a more comprehensive understanding of complex conditions involving multi-organ pathologies [16, 40, 59].

#### 12.2.4 Integration with emerging technologies

The convergence of DTs with other emerging technologies presents exciting possibilities for healthcare innovation [60–62]. Integration with advanced robotics could revolutionize surgical planning and execution through immersive simulations and real-time guidance [45, 60]. Incorporation of blockchain technology might address data provenance and security challenges while facilitating secure information sharing across institutions [104]. Extended reality interfaces could transform how clinicians interact with DTs, enabling intuitive exploration of complex physiological models and collaborative decision-making [62]. The potential synergy between DTs and gene-editing technologies like CRISPR could unlock new approaches for personalized genetic therapies by simulating intervention outcomes before implementation [50–52].

#### 12.2.5 Global health applications

DTs have the potential to address healthcare disparities by enabling resource-efficient approaches to complex medical challenges in low-resource settings [7, 10]. Cloud-based implementations that require minimal local infrastructure could make sophisticated diagnostic and treatment planning tools accessible in underserved regions [28, 104]. Adaptable DT frameworks could be tailored to address region-specific health challenges and populations, potentially transforming global health approaches to infectious diseases, maternal health, and chronic condition management in diverse healthcare systems [6, 9, 13].

The continued evolution of DT technologies in healthcare will demand interdisciplinary collaboration across computational sciences, medicine, engineering, ethics, and policy [1, 2]. As these technologies mature from research tools to clinical applications, addressing the identified challenges while capitalizing on emerging opportunities will be essential for realizing their transformative potential in healthcare.
